# Supplementary material for: A natural constant predicts survival to maximum age
Source: Commun Biol. 2021 May 31;4:641. doi: 10.1038/s42003-021-02172-4 (PMC8166855; doi:10.1038/s42003-021-02172-4)
Supplement: Supplementary file 2 — Supplementary Information [file 42003_2021_2172_MOESM2_ESM.pdf]

Supplementary Information

A natural constant predicts survival to maximum age

Manuel Dureuil*[1, 2], Rainer Froese[3]

[1]Department of Biology, Dalhousie University, 1355 Oxford St, Halifax, B3H 4R2, Canada

[2]Sharks of the Atlantic Research and Conservation Centre, 279 Portland St, Dartmouth, B2Y 1K2, Canada

[3]GEOMAR Helmholtz Centre for Ocean Research, Düsternbrooker Weg 20, 24105 Kiel, Germany

Supplementary Notes

Theoretical maximum age without senescence

Without actuarial senescence, the theoretical maximum age that can be reached by an individual, i.e. the largest observation, would be a logarithmic function of cohort size[1,2]:

$$T_{\max\_NC} = -\frac{0.577 + log_e(N_c)}{M} + t_c. \qquad (S1)$$

where T_{max_NC} is the theoretical maximum age, t_c is the age above which the mortality rate is assumed to be constant, and N_c is the number of individuals alive at age t_c.

Supplementary Tables

Table S1 | Variability in survival to the average maximum age in a cohort across natural populations of wild vertebrates. Confidence intervals (CI) given as extreme (5th and 95th) and central (25th, 50th and 75th) percentiles for *n* species of different vertebrate classes.

Class	n	5th	25th	Median	75th	95th
Actinopterygii	75	0.0013	0.005	0.014	0.033	0.099
Amphibia	15	0.0005	0.003	0.026	0.061	0.129
Aves	64	0.0002	0.001	0.012	0.042	0.113
Elasmobranchii	12	0.0083	0.013	0.020	0.041	0.081
Mammalia	24	0.0026	0.007	0.012	0.032	0.049
Reptilia	12	0.0031	0.008	0.015	0.030	0.041
All	202	0.0004	0.004	0.015	0.038	0.106

Table S2 | Details on literature survival to maximum age for various species. Information is supplementary to Table 1, with comments on how the specific survival to maximum age *P* estimate was obtained and the source reference.

Species	Comment	Source
Laminaria digitata	*P* from life table	Chapman, A.R., 1993. 'Hard'data for matrix modelling of Laminaria digitata (Laminariales, Phaeophyta) populations. Hydrobiologia, 260(1), pp.263-267.
Latrodectus mactans	*P* from life table	Deevey, G.B. and Deevey, E.S., 1945. A life table for the black widow. Trans. Conn. Acad. Arts Sci, 36(1), p.l.
Panope abrupta	*P* from *M* and t_{max}	Noakes, D.J., 1992. On growth and mortality of geoduck clams (Panope abrupta)(or how fast do all 'ducks go to heaven?). Can. Manuscr. Rep. Fish. Aquat. Sci, (2169), pp.22-34.
Panopea abbreviata	*P* from *M* and t_{max}	Zaidman, P.C. and Morsan, E., 2018. Reconstructing populations dynamics: Mortality and recruitment of the southern geoduck Panopea abbreviata. Journal of Sea Research, 135, pp.31-73.
Siliqua patula	mean *P* from life tables	Weymouth, F.W. and McMillin, H.C., 1930. Relative growth and mortality of the Pacific razor clam (Siliqua patula, Dixon) and their bearing on the commercial fishery. Bull. Bur. Fish., Wash., 1099, pp.543-567.
Spisula polynyma	*P* from *M* and t_{max}	Hughes, S.E. and Bourne, N., 1981. Stock assessment and life history of a newly discovered Alaska surf clam (Spisula polynyma) resource in the southeastern Bering Sea. Canadian Journal of Fisheries and Aquatic Sciences, 38(10), pp.1173-1181.
Callinectes sapidus	*P* from *M* and t_{max}	Hewitt, D.A., Lambert, D.M., Hoenig, J.M., Lipcius, R.N., Bunnell, D.B. and Miller, T.J., 2007. Direct and indirect estimates of natural mortality for Chesapeake Bay blue crab. Transactions of the American Fisheries Society, 136(4), pp.1030-1040.
Cerithidea decollata	*P* from *M* and t_{max}	Cockcroft, V.G. and Forbes, A.T., 1981. Growth, mortality, and longevity of Cerithidea decollata (Linnaeus) (Gastropoda, Prosobranchia) from Bayhead mangroves, Durban Bay, South Africa. Veliger, 23(4), pp.300-308.
Aedes aegypti	*P* from *M* and t_{max} (females)	McDonald, P.T., 1977. Population characteristics of domestic Aedes aegypti (Diptera: Culicidae) in villages on the Kenya Coast I. Adult survivorship and population size. Journal of Medical Entomology, 14(1), pp.42-48.
Apis mellifera	median *P* from life tables	Sakagami, S.F. and Fukuda, H., 1968. Life tables for worker honeybees. Population Ecology, 10(2), pp.127-139.
Cyperus rotundus	Predicted *P*	Neeser, C., Aguero, R. and Swanton, C.J., 1997. Survival and dormancy of purple nutsedge (Cyperus rotundus) tubers. Weed Science, pp.784-790.
Phlox drummondii	*P* from life table	Leverich, W.J. and Levin, D.A., 1979. Age-specific survivorship and reproduction in Phlox drummondii. The American Naturalist, 113(6), pp.881-903.
Various tree species	Assumed *P*	Botkin, D.B., Janak, J.F. and Wallis, J.R., 1972. Some ecological consequences of a computer model of forest growth. The Journal of Ecology, pp.849-872.
Homo sapiens	median *P* from life tables of Japanese males and females (1776-1795)	Jannetta, A.B. and Preston, S.H., 1991. Two centuries of mortality change in central Japan: the evidence from a temple death register. Population Studies, 45(3), pp.417-436.
Archaster angulatus	Predicted *P*	Keesing, J.K., 2018. Rate of natural mortality in the sea star Archaster angulatus (Echinodermata: Asteroidea). Journal of the Marine Biological Association of the United Kingdom, 98(7), pp.1689-1693.

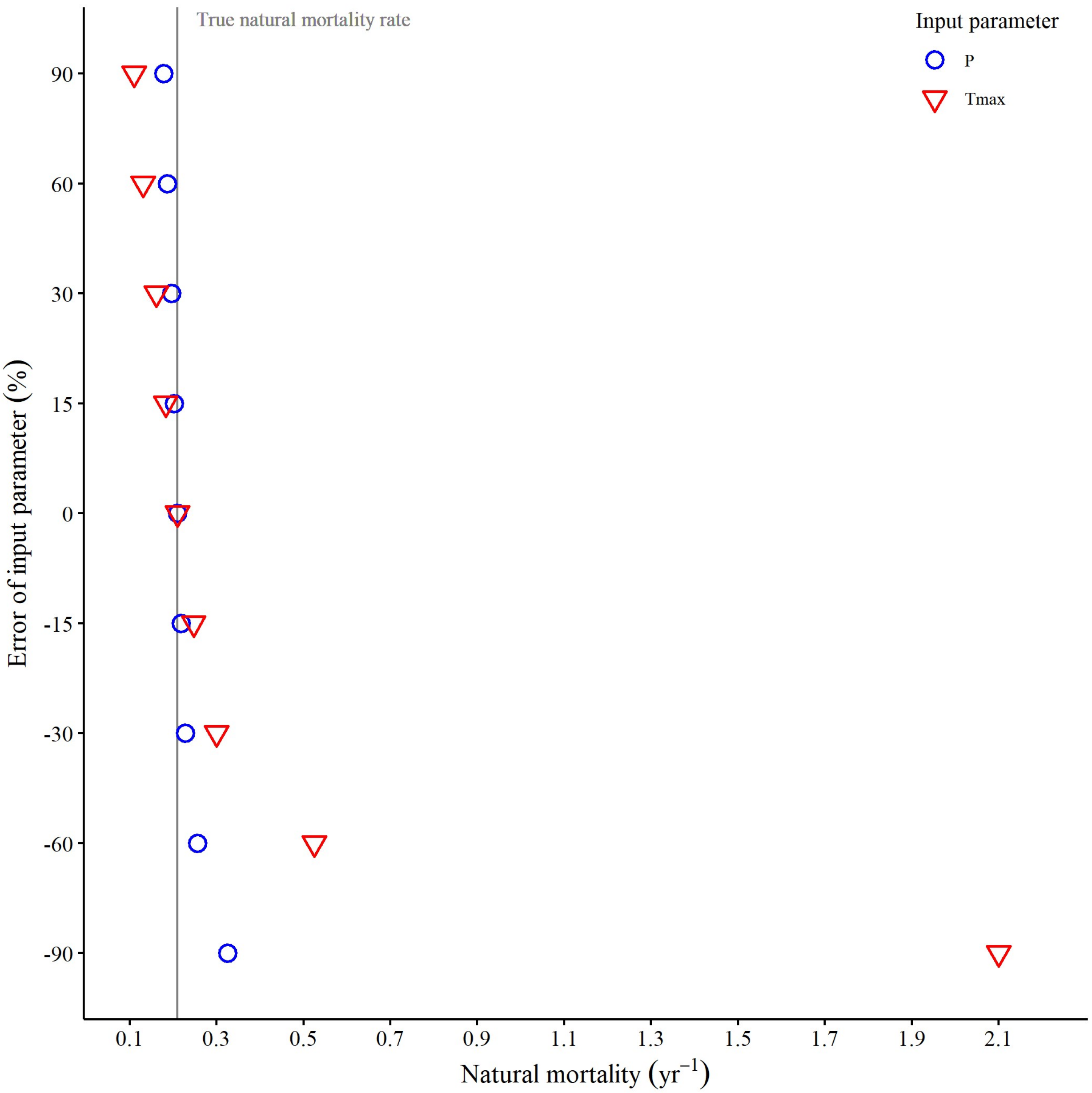

Figure S1 | Effect of incorrect input parameters on the estimate of the average adult natural mortality rate. The input parameters for the average adult natural mortality rate, *M*, (see manuscript equation (4)) are maximum age (red triangles) and the proportion surviving from birth to maximum age (blue circles).

Supplementary References

1. Holt, S. J. A Note on the Relation Between the Mortality Rate and the Duration of Life in an Exploited Fish Population. *Int. Comm. Northwest Atl. Fish. Res. Bull.* **2**, 73–75 (1965).
2. Hoenig, J. M. Should Natural Mortality Estimators Based on Maximum Age Also Consider Sample Size? *Trans. Am. Fish. Soc.* **146**, 136–146 (2017).
