# Supplementary material for: A natural constant predicts survival to maximum age
Source: Commun Biol. 2021 May 31;4:641. doi: 10.1038/s42003-021-02172-4 (PMC8166855; doi:10.1038/s42003-021-02172-4)
Supplement: Supplementary file 3 — Descriptions of Additional Supplementary Files [file 42003_2021_2172_MOESM3_ESM.pdf]

Descriptions of Additional Supplementary Files

Supplementary data 1
Description: For each of the examined vertebrate species, with taxonomic class, scientific name and common name, the proportion of individuals surviving to the average maximum age in a cohort P is provided as calculated from the adult natural mortality rate M and average maximum age tmax. Information on the source reference from which M and tmax values were taken is given.
