# Supplementary material for: A natural constant predicts survival to maximum age
Source: Commun Biol. 2021 May 31;4:641. doi: 10.1038/s42003-021-02172-4 (PMC8166855; doi:10.1038/s42003-021-02172-4)
Supplement: Supplementary file 5 — Reporting Summary [file 42003_2021_2172_MOESM5_ESM.pdf]

nature research

Corresponding author(s): Manuel Dureuil

Last updated by author(s): Apr 15, 2021

Reporting Summary

Nature Research wishes to improve the reproducibility of the work that we publish. This form provides structure for consistency and transparency in reporting. For further information on Nature Research policies, see our Editorial Policies and the Editorial Policy Checklist.

Statistics

For all statistical analyses, confirm that the following items are present in the figure legend, table legend, main text, or Methods section.

n/a	Confirmed	
☐	☒	The exact sample size (n) for each experimental group/condition, given as a discrete number and unit of measurement
☐	☒	A statement on whether measurements were taken from distinct samples or whether the same sample was measured repeatedly
☐	☒	The statistical test(s) used AND whether they are one- or two-sided<br>*Only common tests should be described solely by name; describe more complex techniques in the Methods section.*
☐	☒	A description of all covariates tested
☒	☐	A description of any assumptions or corrections, such as tests of normality and adjustment for multiple comparisons
☐	☒	A full description of the statistical parameters including central tendency (e.g. means) or other basic estimates (e.g. regression coefficient) AND variation (e.g. standard deviation) or associated estimates of uncertainty (e.g. confidence intervals)
☒	☐	For null hypothesis testing, the test statistic (e.g. F, t, r) with confidence intervals, effect sizes, degrees of freedom and P value noted<br>*Give P values as exact values whenever suitable.*
☒	☐	For Bayesian analysis, information on the choice of priors and Markov chain Monte Carlo settings
☒	☐	For hierarchical and complex designs, identification of the appropriate level for tests and full reporting of outcomes
☒	☐	Estimates of effect sizes (e.g. Cohen's d, Pearson's r), indicating how they were calculated

Our web collection on statistics for biologists contains articles on many of the points above.

Software and code

Policy information about availability of computer code

Data collection	Data was collected from open sources with references and links provided in the Materials and Methods section
Data analysis	R open source statistical software was used to analyse the data

For manuscripts utilizing custom algorithms or software that are central to the research but not yet described in published literature, software must be made available to editors and reviewers. We strongly encourage code deposition in a community repository (e.g. GitHub). See the Nature Research guidelines for submitting code & software for further information.

Data

Policy information about availability of data

All manuscripts must include a data availability statement. This statement should provide the following information, where applicable:

- Accession codes, unique identifiers, or web links for publicly available datasets
- A list of figures that have associated raw data
- A description of any restrictions on data availability

The data that support the findings of this study are based on literature reviews and therefore publicly available. The data on the survival to maximum age per species will be provided as electronic supplement.

Field-specific reporting

Ecological, evolutionary & environmental sciences study design

All studies must disclose on these points even when the disclosure is negative.

Study description	A comprehensive literature review was conducted on vertebrate's natural survival and maximum age, with additional selected examples from other taxa, to compare the proportions surviving to average maximum age in a cohort across different groups of living beings and to derive a practical application for the estimation of the mean adult natural mortality rate. The median proportions surviving to the average maximum age in a cohort were then compared using graphical analysis (box-plots) and statistical analysis (Kruskal-Wallis rank sum test). Evolutionary theory was used to explain the observations.
Research sample	Samples where taken from a comprehensive literature review based on existing datasets. The links for online datasets and the references for published datasets are provided in the Materials and Methods section.
Sampling strategy	Samples where based on a comprehensive literature review from existing datasets providing already summarized information for different taxonomic classes.
Data collection	Literature review
Timing and spatial scale	We did not restrict time or spatial scale
Data exclusions	Cases considered unlikely where excluded form the analysis with a detailed justification and description in the Materials and Methods section. However, a sensitivity analysis was done comparing the unlikely cases removed dataset with the full dataset, and no significant difference for the main results between the datasets was found.
Reproducibility	The data is provided in the Supplementary Information. The links and references to the raw data are provided as well. A detailed description is given explaining the preparation of the raw data and the analysis. The graphical and statistical analysis can be reproduced using open source software.
Randomization	Organisms were grouped according to taxonomic classes.
Blinding	Blinding was not relevant for our study, because all data was assigned based on taxonomy

Did the study involve field work? ☐ Yes ☒ No

Reporting for specific materials, systems and methods

We require information from authors about some types of materials, experimental systems and methods used in many studies. Here, indicate whether each material, system or method listed is relevant to your study. If you are not sure if a list item applies to your research, read the appropriate section before selecting a response.

Materials & experimental systems

n/a	Involved in the study	
☒	☐	Antibodies
☒	☐	Eukaryotic cell lines
☒	☐	Palaeontology and archaeology
☐	☒	Animals and other organisms
☒	☐	Human research participants
☒	☐	Clinical data
☒	☐	Dual use research of concern

Methods

n/a	Involved in the study	
☒	☐	ChIP-seq
☒	☐	Flow cytometry
☒	☐	MRI-based neuroimaging

Animals and other organisms

Policy information about studies involving animals; ARRIVE guidelines recommended for reporting animal research

Laboratory animals	Study did not involve laboratory animals.
Wild animals	This study did not sample wild animals itself. Information on wild animals were based on a literature review and existing datasets.
Field-collected samples	This study did not involve sample collected from the field.
Ethics oversight	No ethical approval or guidance was required because all information was taken form the literature and no animals were sampled directly for this study.

Note that full information on the approval of the study protocol must also be provided in the manuscript.
